# Green Palladium Nanoparticles: Mechanism of Synthesis and Biomedical Application

**DOI:** 10.3390/jfb17030152

**Published:** 2026-03-19

**Authors:** Ekaterina O. Mikhailova

**Affiliations:** Institute of Innovation Management, Kazan National Research Technological University, K. Marx Street 68, 420015 Kazan, Russia; katyushka.glukhova@gmail.com

**Keywords:** palladium nanoparticles, green synthesis, mechanism of synthesis, antibacterial activity, anticancer activity

## Abstract

Green synthesis of nanoparticles has become one of the most popular research areas in recent decades due to its environmentally friendly nature and the minimization of harmful chemical by-products. This review focuses on the mechanism of palladium nanoparticle (PdNP) biosynthesis using bacteria, fungi, algae, and plants, and their potential biological activities, such as antibacterial, anticancer, antioxidant, and other properties, with the aim of their further biomedical applications. The role of various biomolecules in these processes is also discussed.

## 1. Introduction

The beginning of the 21st century witnessed a surge in research interest surrounding the synthesis of metallic nanoparticles (NPs). These remarkable entities ushered in a new era of nanotechnology advancement. Early methods for creating metal nanoparticles relied on physical and chemical processes, which, despite their widespread use, were often expensive, laborious, and energy-consuming. More significantly, these techniques carried inherent risks to the environment and living organisms. Another path forward was “green” nanotechnology, offering the potential to develop non-toxic goods, reduce waste and energy usage, and transform damaging industries. In recent decades, it has become clear that biological systems (bacteria, fungi, and plants) are capable of converting metal ions into nanoparticles due to reducing processes in their own biosystems, using a variety of metabolites [1,2]. As a result of this “green technology,” nanoparticles with exceptional properties are now being synthesized and manufactured, holding great promise for biomedical applications such as treating and preventing a wide spectrum of human diseases.

Silver and gold nanoparticles are now the most popular among researchers, but the study of other materials is also gaining momentum. One such nanoparticle that attracts scientists’ interest is palladium nanoparticles (PdNPs). The discovery of palladium, a transition metal in the platinum group, occurred in 1803 [3,4] by the English scientist William Hyde Wollaston. Industrial production of palladium began in the 20th century. In the 20th century, palladium began to be produced industrially. It was used as a catalyst in the chemical industry, for electroplating, and in jewelry. Palladium nanoparticles, due to their unique electronic and chemical properties, have attracted special attention as heterogeneous nanocatalysts for various organic transformations, such as reactions of C-C compounds, hydrogenation of alkenes and alkynes, oxidation reactions, reduction of nitroarenes, and decomposition of dyes [5].

Due to its biological inertness, it is used for the manufacture of medical instruments, pacemakers, and dentures, and also in the form of complex compounds for the creation of anti-cancer drugs [5,6,7,8,9]. The characteristics of palladium make the produced nanoparticles potentially valuable for practical use in medical applications. The antibacterial, antifungal, anticancer, antioxidant, and other properties of PdNPs have been discovered [1,2,3,4,5,6,7,8,9]. In this regard, green methods for producing PdNPs with a wide range of biological objects are becoming increasingly important. The characteristics of PdNPs derived from specific sources and their biological properties can be greatly influenced by various cellular components, such as proteins, enzymes, and acids. This gives rise to a plethora of techniques employed in the nanoparticle characterization—the shape and size of synthesized “green” PdNPs are defined by scanning electron microscopy (SEM) and transmission electron microscopy (TEM), scanning electron microscopy (SEM)—to evaluate the morphology of palladium nanoparticles, UV/Vis spectrophotometry, and dynamic light scattering (DLS)—to evaluate the physical properties. FTIR analysis (Fourier transform infrared spectroscopy) is able to identify biomolecules that are involved in reducing palladium ions and stabilizing nanoparticles [8]. The crystallinity of the nanoparticles was calculated based on X-ray diffraction measurements [9]. The phases of green synthesis, the influence of physicochemical factors, and the participation of various compounds of biological origin require careful consideration both for understanding the essence of biosynthesis and for its potential scaling up in industrial conditions. The concept of “green synthesis” is significant because it can be understood in two interconnected ways: on the one hand, it implies environmental friendliness, and on the other, the non-toxic effect on healthy human cells and the targeted action of nanoparticles. Therefore, the biosynthesis of palladium nanoparticles, achieved through the “green” method, is of particular interest as a promising avenue for the development of safe drugs for the treatment of a wide range of diseases. This review focuses on the mechanism of palladium nanoparticle synthesis by living organisms such as bacteria, fungi, algae, and plants, and explores potential applications in biomedicine.

## 2. PdNP Biosynthesis and Its Mechanism

Nanotechnology techniques were developing rapidly at the end of the 20th century and the beginning of the 21st century. Palladium nanoparticles were no exception to this trend. There are two main approaches used to produce nanoparticles: the “bottom-up” and “top-down” strategies [10]. With the “top-down” strategy, bulk material is crushed into small particles by reducing the size using various physical and chemical methods, while “bottom-up” nanoparticles are synthesized via a self-assembly of atoms into nuclei, which further develop into nanoscale particles. It includes both chemical and biological methods [10]. The applied physical methods, such as physical vapor deposition, magnetron sputtering, and laser ablation, and chemical methods, like electrochemical deposition, sonochemical preparation, the sol–gel method, and supercritical fluid nucleation, have several important disadvantages. These methods are energy-intensive and require maintaining high temperatures and pressures. Additionally, they frequently use harmful solvents, reducing agents, and stabilizers, which can pose a threat to the environment and living organisms.

It is no coincidence that biological methods for synthesizing nanoparticles have gained such popularity. Strategies for green nanoparticle synthesis rely on the fundamental principles of green chemistry, which were formulated back in 1998 [11]. The transition to renewable raw materials (1), the use of low-temperature technologies (2), the possibility of eliminating unnecessary stages thanks to the catalytic basis of the process (3), and the biodegradability of produced by-products (4) and minimization of the use of harmful auxiliary substances (5) become possible with the use of living organisms, and the products of their vital activity for the synthesis of metal nanoparticles. Nanoparticle biosynthesis is considered a promising alternative, enabling the production of nanoparticles from biological materials at a rapid, efficient, and cost-effective level. Importantly, the biomolecules that play a crucial role in the synthesis process, as reducing agents and stabilizers, are consistent with the green agenda, reflecting the desire for an environmentally friendly approach.

Furthermore, it is believed that by biological synthesis, the physical properties of nanoparticles, including their shape and size, can be controlled [12]. The development of efficient biosynthesis protocols can not only simplify the synthesis process but also increase the possibilities for its modification. This makes PdNPs more suitable for conjugation with antibodies and ligands, various drug delivery, and for diagnostic imaging [13].

Bacterial, fungal, and plant cells produce a vast array of low- and high-molecular-weight biocompounds (acids, alcohols, polysaccharides, polyphenolic compounds, proteins, etc.), which, due to their redox potential, are capable of reducing metal salt cations found in water and soil. They are capable of serving as reducing agents, as well as stabilizers and capping agents, for metal nanoparticles such as palladium.

**Figure 1:**
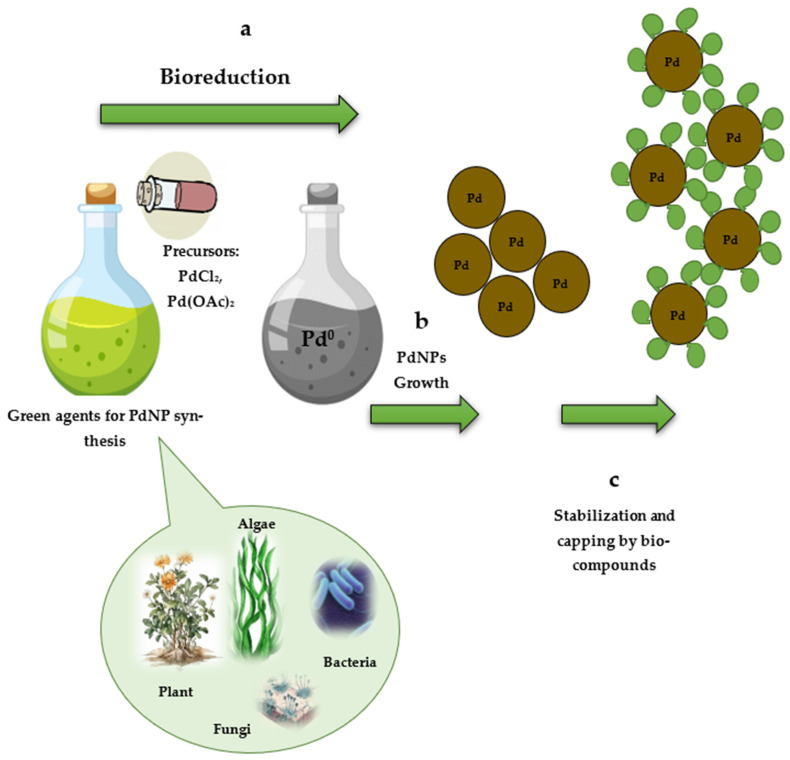
The proposed mechanism of palladium nanoparticles synthesis. (**a**) reduction by different biological sources; (**b**) nanoparticle’s growth; (**c**) stabilization and capping by plant, fungal or bacterial compounds.

The process of PdNP biosynthesis requires the participation of two main components: a precursor (a solution of palladium salts) and a biological extract (plant, bacterial, fungal, and algal biomass), which are mixed most often at room temperature, triggering the biosynthesis of nanoparticles. The reaction medium color changing to dark brown or black indicates their formation [4]. Although the exact mechanism of palladium nanoparticle biosynthesis has not been fully understood, the process generally follows a pattern typical of most other metal nanoparticle syntheses [14]. Bioreduction and metal ion nuclei are formed during the initial phase of activation (1), where metal ions are reduced to a state of zero oxidation (Figure 1) [15]. This process involves biological molecules that can reduce metal ions by transferring electrons. The subsequent growth and agglomeration of small palladium particles into larger and thermodynamically more stable entities occurs during the second phase (2). The process culminates in the third phase, resulting in the formation of various PdNP configurations, which can take the form of spheres, rods, triangles, wires, pentagons, or hexagons (3). These nanoparticles are stabilized through the presence of diverse biomolecules, such as proteins, polyphenols, and alcohols. Cellular biomolecules contain functional groups binding to metals and also act as capping agents that can direct or inhibit the growth of nanoparticles by controlling their size [4]. In addition, capping agents can mediate the biological and potentially beneficial medical properties of palladium nanoparticles. Parameter optimization, such as the metal salt concentration, temperature, and pH of the reaction medium; the extract addition rate and contact time; and the concentration of the biological substrate used as a “factory” for PdNP biosynthesis, is necessary to control the shape, size, and crystallinity of the resulting palladium nanoparticles. The multitude of biological reducing agents, stabilizers, and capping agents from living organisms provides a wide range of opportunities for experimentation in the synthesis and potential green nanoparticle applications.

### 2.1. By Bacteria

Bacteria are not the most common biofactory for metal nanoparticle production. The difficulties associated with choosing conditions for culturing microorganisms for efficient biosynthesis of palladium nanoparticles do not seem to encourage a significant increase in research on this topic.

Cultivation techniques are essential when working with microorganisms. Disadvantages of microbial nanoparticle synthesis include the need for specialized equipment for culturing microorganisms and for monitoring and maintaining this process; variability of the microbial product depending on cultivation conditions; and, finally, biosafety issues that may arise with certain microbial strains.

**Figure 2:**
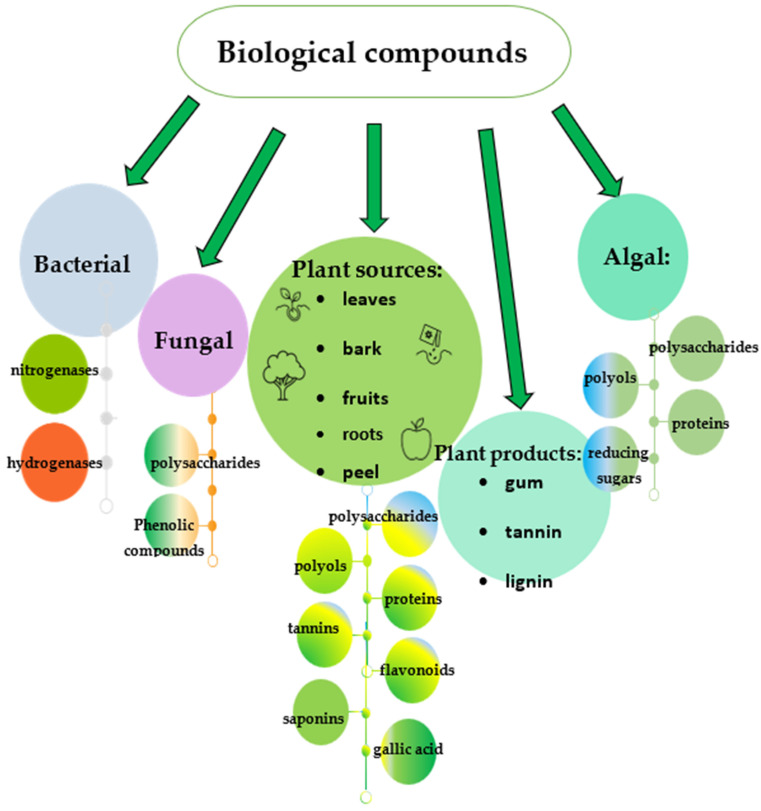
Reducing bio-compounds involved in green PdNP synthesis.

Initially, the bacteria adsorb Pd^2+^ ions due to the presence of a large number of negatively charged groups on the cell surface. Enzymes seem to play a key role in bacterial biosynthesis, as is the case with other metal nanoparticles. Periplasmic hydrogenases have been found to be involved in the reduction of Pd^2+^ to Pd^0^ in bacteria from the genus *Shewanella* [16]. Cyanobacteria are thought to involve two groups of enzymes—nitrogenases and hydrogenases. For instance, *Anabaena variabilis* produces two distinct nitrogenases: one operates only in heterocysts under anaerobic circumstances, while the other works in both heterocysts and vegetative cells but only under these conditions. Additionally, hydrogenase is capable of reducing hydrogen ions to molecular hydrogen in addition to other electron acceptors. The ferredoxins in thylakoids serve as a connection between hydrogenase and various electron donors and acceptors in both photosynthetic and non-photosynthetic systems. Both nitrogenase and hydrogenase can act as reducing agents for some metal salts [17]. Fundamentally different *Desulfovibrio desulfuricans* NCIMB 8307 and *Bacillus benzeovorans* produce similar monodisperse intracellular PdNPs, with those formed by formate oxidation being larger (5–7 nm) than those formed by hydrogen oxidation (1–4 nm). When hydrogen was used as an electron donor, hydrogenases most likely played a dominant role (along with other enzymes and biomolecules that generate electrons) in determining the nature of the final PdNPs in *D. desulfuricans* strains. However, another mechanism employing formate dehydrogenase is apparently involved in the oxidation of formate. At the same time, regardless of the specific enzymatic mechanism, the oxidation of formate provides a source of electrons necessary for bacteria to convert Pd^2+^ into nanoparticles [18]. *Bacillus thuringiensis* reduces palladium to PdNPs using endogenous electron donors. Transcriptome analysis revealed differentially expressed genes responding to palladium treatment under anaerobic and aerobic conditions. It was found that the genes encoding NADH-quinone oxidoreductase, dehydrogenases, cytochrome c reductase, cytochrome c oxidase, quinone cycle, and ribE in *B. thuringiensis* Y9 have a strong positive relationship with palladium reduction. It has been established that hydrogenase also plays a vital role in the bioreduction process [19]. The periplasmic cytochrome c3 and hydrogenases are involved in the electron-transfer processes for lactate oxidation and for the initial reduction of Pd^2+^ ions, and hydrogenases act as nucleation sites in *D. desulfuricans* [20]. NADH dehydrogenase and hydrogenase (HydA and HyaB) participate in the production of bio-PdNPs using formate as an electron donor in *S. oneidensis* [21]. Two pathways for PdNP formation in *S. oneidensis* are proposed, using formate as the electron donor. Pathway I involves formate dehydrogenases (FDH), NADH dehydrogenase, a quinone pool, and cytochromes. Pathway II relies on FDH and hydrogenases. While PdNPs produced via Pathway I are localized on the outer membrane, both pathways can contribute to nanoparticle formation in the periplasmic space [21]. In *Geobacter sulfurreducens*, cytochromes are suggested to be involved in the extracellular reduction of palladium ions to form nanoparticles [22]. The involvement of various biocompounds in the synthesis process is illustrated in Figure 2. The above outlines the main pathways for the biosynthesis of palladium nanoparticles using bacteria. The enzymatic pathway appears to be a very promising approach, as it can be implemented using either the bacteria themselves or isolated or synthesized enzymes capable of biosynthesis. Furthermore, the development of bioengineered microorganisms with high enzyme activity involved in PdNP biosynthesis could facilitate the scaling of the process and the production of nanoparticles under industrial conditions. Prospective properties of the genetically engineered organisms can include enhanced resistance to metal toxicity, high throughput, energy efficiency and ecological procedures [23]. To increase enzyme activity, many cultivation parameters must be optimized, including nutrition, light intensity, pH of the medium, temperature, stirring speed, and buffer concentration.

At the same time, there are apparently other, alternative non-enzymatic pathways for bacterial synthesis of PdNPs. Thus, no hydrogenase mechanism of PdNP synthesis was found in *Cupriavidus necator*, as palladium nanoparticles continued to form in both pasteurized cells and autoclaved cells with inactive hydrogenases [24]. Although active enzymes do not appear to be required, it is possible that coordination of Pd^2+^ with chemical groups on the cell surface helps initiate the repair process, but this requires further study.

Bacterial biosynthesis is possible both intracellularly and extracellularly. The latter option seems preferable from the point of view of receiving the final product, because it does not require cell destruction and additional purification steps. Synthesis was conducted in the periplasm or extracellular space of *S. oneidensis*, while no PdNPs were detected in the bacterial cytoplasm [25]. A contrasting scenario has been observed in the cyanobacteria *Anabaena* and *Calothrix*, where metal nanoparticles synthesized intracellularly are subsequently released into the culture medium through canal-like intercytoplasmic connections [17]. Extracellular synthesis was found in the cyanobacterium *Plectonema boryanum* [26]. Meanwhile, the intracellular production of palladium nanoparticles was demonstrated in two anaerobic strains of *D. desulfuricans* and one aerobic strain of *Bacillus benzeovorans*, utilizing hydrogen and formate as electron donors [18]. According to TEM studies, the majority of the PdNPs produced by *B. megaterium* were found in the periplasmic space, where they formed crown-like structures on the cell [27]. The localization of biosynthesis may also be linked to the enzymes that drive this process: for example, hydrogenases are localized in the cell membrane, and in the case of *D. desulfuricans*, in the periplasmic space [28]. A comparable distribution is observed in *S. oneidensis*, where NADH dehydrogenases facilitate the formation of PdNPs on the outer membrane, whereas hydrogenases—particularly HyaB—are responsible for nanoparticle synthesis in the periplasm [21].

It is interesting to note that the extracellular electron transfer components, the MtrC outer membrane cytochrome and soluble redox shuttles (flavins), have a significant impact on the PdNPs’ formation in these bacteria. By regulating gene expression and adding exogenous substances, these component concentrations in the bacterial environment can be controlled, leading to significant changes in the rate of nanoparticle synthesis, their size, and their cellular localization. The increased flavin concentration alters nanoparticle dimensions through binding and nucleation interactions [21].

A key factor is the ability of capping agents to endow palladium nanoparticles with specific properties, thereby increasing their potential for medical applications. Furthermore, integrating palladium nanoparticles into organic matrices can enhance their stability and regulate their size. It was found that carbonyl groups present in enzymes and proteins secreted by microbial cells can aid in stabilizing biologically synthesized nanoparticles. Additionally, functional groups such as –OH, –SH, –COOH, and –NH_2_ in microbial proteins are crucial for reducing metals and stabilizing the resulting nanoparticles [29]. For instance, it has been shown that PdNPs produced by cyanobacteria are released into the culture medium encased in a polysaccharide shell [17]. This phenomenon is likely explained by the metal-binding properties of various cyanobacterial components. Carboxyl groups, polyphosphates, and amino acids within the cells are thought to facilitate metal binding. Furthermore, polysaccharides, both within the cell wall and surrounding it as water-soluble polymers, offer a high density of sites for metal ion attachment. In addition, the majority of cyanobacterial polysaccharides have high uronic acid subunits, which are also capable of efficiently binding metal ions by their carboxyl groups. With a variety of functions, including antibacterial, antioxidant, anticancer, anti-inflammatory, antiviral, and immunomodulatory, they can enhance the clinical effect of nanoparticles [30]. For example, such PdNPs in combination with bacterial polysaccharides with antimicrobial properties may be better candidates for combating polymicrobial infections. Another possible mechanism of metal binding in cyanobacteria is the formation of metallothioneins or metal-binding proteins. These proteins bind metal ions in the form of metal thiolates and often contain a characteristic set of sulfur-containing amino acids [26].

The shape and size of nanoparticles are of great importance, especially for subsequent practical applications. The bacteria-synthesized PdNPs have different sizes but are usually spherical (Table 1).

The size and shape of particles may be affected by the concentration of metallic salts and the pH of the medium. The initial concentrations of Pd^2+^ also play a role in the size-controlled formation of NPs.

A comparison of chemical and biological synthesis methods using *Citrobacter* sp. revealed that the resulting bionanoparticles were smaller in size than chemically produced ones. The presence of the microbial cells increases the nucleation sites for PdNPs deposition and promotes crystal growth, which is self-sustaining due to the autocatalytic reduction of more Pd^2+^. This leads to a decrease in the nanoparticle size, while the nucleation rate plays an important role in the particle size formed. The higher the nucleation rate, the smaller the size of the nanoparticles was found [27]. Interesting data were found during the PdNP synthesis using mutant strains of *S. oneidensis*: the high efficiency of nanoparticles as catalysts is due to the smallest particle size and the presence of many functional groups. Synthesis of such nanoparticles occurred in *Shewanella* strains, which do not have the ability to form biofilms, because the absence of biofilm can minimize metal agglomeration, resulting in uniform particle size dispersion [32].

An important factor controlling the size of biosynthesized nanoparticles is synthesis localization. For example, *Cupriavidus metallidurans*-derived PdNPs synthesized through intracellular pathways tend to be smaller than those produced extracellularly [31]. Variations in the activity and localization of enzymes and membrane proteins involved in “Pd^2+^ trafficking,” which facilitates the formation of Pd^0^ nuclei, can influence the size, distribution, and localization of PdNPs. This phenomenon was observed in the biosynthesis processes involving *Acidocella aromatica* and *Acidiphilium cryptum* [33]. Additionally, evidence suggests that living bacterial cells, as opposed to dead cells, are essential for both the formation and stabilization of PdNPs [33,34]. Extracellular PdNPs from *Geobacter sulfurreducens* were smaller in size, averaging 14 ± 3 nm in diameter, while cell surface-bound PdNPs were larger in size, averaging 25 ± 11 nm [35]. Intriguingly, the size of the Pd^0^ clusters was apparently restricted in the z-dimension during biosynthesis, which resulted in the creation of particles with a maximum size of 20–30 nm in this direction. This implies that the growth of Pd^0^ accumulating in the periplasmic space can be determined by the distance between the inner and outer cell membranes [23].

Moreover, it is shown that with an increase in the ratio of cell cry weight to Pd^2+^ from 3:1 to 9:1, smaller and more dispersed particles were gradually obtained. PdNPs are not only associated with the cell surface but also located inside the periplasm by *G. sulfurreducens*. From the other side, the already formed PdNPs could also act as a nucleation site to autocatalytically reduce and precipitate more palladium on their surface [35].

An important characteristic is also the magnitude of the zeta potential characterizing the nanoparticle stability. A higher value of the zeta potential results in greater stability, which in turn leads to smaller particle formation. It is assumed that some biomolecules capped the NPs, causing a negative charge over them and leading to their stabilization [31]. The pH of the medium was also important because a higher zeta potential was observed during bioreduction at pH 8 compared to pH 6 [31]. Furthermore, the physicochemical characteristics of nanoparticles play an important role in their use as safe, effective, and stable nanocarriers in drug delivery systems. Homogeneous (monodisperse) nanoparticles are preferred for in vitro and in vivo applications.

It should also be noted that cellular biomolecules (e.g., proteins) that contain functional groups are capable of acting as a capping agent, directing or inhibiting the growth of nanoparticles and controlling their size and function [36].

### 2.2. By Fungi

The application of fungi in the production of palladium nanoparticles is comparatively uncommon. Nevertheless, among the fungal species that are utilized, yeasts are the most frequently employed for biosynthesis. Fungi excrete many extracellular reductive enzymes, which makes them good candidates for extracellular NPs production. Enzymatic fungal biosynthesis has already been described for other metal nanoparticles [13]; however, the literature data on fungal biosynthesis of PdNPs rather indicate other synthesis mechanisms. Furthermore, the insufficient study of this issue requires further research into the mechanism of fungal biosynthesis.

Using the fungus *Candida krusei*, spherical palladium nanoparticles with a size of less than 50 nm were synthesized. The FTIR spectra of the polymer evidenced the presence of carboxyl groups, which may serve as binding sites for divalent cations [37]. A mixture of sherical and triangular shapes with a size of 32 nm was detected for *Saccharomyces cerevisiae*-PdNPs [38]. The bioactive functional group, such as hydroxyl and other functional groups, stabilizes the PdNPs in the mixture, and phenolic compounds are involved in the stabilization and capping process [38]. The baker’s yeast *S. cerevisiae* reduced Pd^2+^ ions at room temperature. The produced nanoparticles were located on the surface of yeast cells, and the surface of *S. cerevisiae* was coated with biological molecules containing functional groups capable of adsorbing Pd^2+^ ions in an aqueous solution at pH 1.0. The process can be divided into two stages: (1) absorption of Pd^2+^ ions from solution by yeast cells and (2) bio-reduction of Pd^2+^ ions into metallic Pd^0^ with formate as an electron donor under anaerobic conditions, because *S. cerevisiae* cells apparently can oxidize formate and transfer two electrons to Pd^2+^ ions [39]. PdNPs were almost uniformly distributed in yeast cells, including the cell wall and cytoplasm, indicating that Pd ions may not have a strong interaction with cellular materials [40].

Another example is the biosynthesis of PdNPs based on the edible mushroom *Agaricus bisporus*, often used in homeopathic medicine due to its biologically active components, such as carbohydrates, proteins, dietary fiber, riboflavin, niacin, iron, pantothenic acid, and amino acids, and phenols, flavonoids, alkaloids, and terpenoids [41]. Given the diverse biological activities of fungal extracts—including antiviral, antibacterial, antiparasitic, anti-inflammatory, cardiovascular, and antidiabetic effects—the resulting nanoparticles can exhibit such properties due to the presence of capping agents derived from these extracts. FTIR analysis of the biogenic PdNPs indicated that polysaccharides, amides, and phenolic acids served as reducing agents and were also responsible for the biostabilization of the nanoparticles (Figure 2). The resulting nanoparticles had a triangular shape and a size of about 15.6 nm [41]. Extracts of the chaga mushroom (*Inonotus obliquus*), valued in traditional Russian medicine for its antiproliferative, anticancer, antioxidant, anti-inflammatory, antiviral, and antibacterial effects, have also been reported to facilitate the biosynthesis of PdNPs [42]. The FTIR analysis testified that amine compounds with aliphatic structures in chaga extract were dominantly adsorbed on PdNPs, implying they acted as a surfactant during the synthesis of spherical PdNPs at low concentrations of chaga extract. However, an increase in concentration led to changes indicating that the compounds in the chaga extract, representing carboxylic acid and hydroxyl groups, were coordinated with the PdNPs’ surface and then replaced the amino compounds adsorbed on the surface. This indicates that the phenolic acid constituents of the chaga extract actively participate in the formation of porous palladium nanoparticles. High zeta-potential values may be associated with an increase in surface area caused by the formation of a rough surface morphology and porous nanostructure during the adsorption of a large amount of chaga extract substances, which create a negative charge. Such a negatively charged surface of chaga-PdNPs can be used for the delivery of positively charged therapeutic molecules through electrostatic interactions [42].

Thus, bacterial and fungal synthesis can be different in nature and involve different cellular mechanisms and biomolecules. PdNPs synthesized and capped with fungal polysaccharides may be a promising approach to treating various diseases. These polysaccharides possess antidiabetic, antioxidant, antiviral, antilipidemic, antitumor, and immunomodulatory potential. For instance, fungal polysaccharides can effectively reduce blood glucose levels in Streptozotocin-induced diabetic mice; some studies have demonstrated their efficacy in significantly reducing total cholesterol [43]. Extracellular synthesis is also preferable due to mediation by secreted metabolites, since it leads to the accumulation of nanoparticles on the surface of fungal cells or in the culture medium [38,44]. pH, temperature, and the composition of the culture medium play a key role in determining the size, shape, and stability of nanoparticles. Fungi can exhibit varying responses to changing pH values, which affects the oxidation-reduction potential and biological compounds involved in nanoparticle synthesis. Temperature can influence the reaction rate and kinetics of nanoparticle formation. The concentration of the starting materials in the culture medium is another important factor that dictates both the yield and the size of the resulting nanoparticles [45]. Furthermore, the individual enzymatic mechanisms and metabolic pathways of each fungal species determine the diversity of nanoparticle morphology and size. However, the evaluation and development of palladium nanoparticle biosynthesis require further study.

### 2.3. By Algae

The synthesis of palladium nanoparticles can be facilitated by algal extracts, which serve as effective reducing and capping agents. The intrinsic presence of pharmacologically active metabolites (including alkaloids, flavonoids, and terpenoids) makes algae a highly attractive platform for the generation of biocompatible metal nanoparticles. Cubical, spherical, and truncated triangular PdNPs were synthesized by the green algae *Botryococcus braunii* [46]. The synthesized PdNPs were about 5 nm in size. Functional group analysis of *B. braunii* revealed that proteins, polysaccharides, amides, and long-chain fatty acids serve as the primary agents for the reduction, capping, and stabilization of palladium nanoparticles (Figure 2) [46]. At the same time, saccharides and other phosphate-containing compounds, and the -OH group of polyols, play a role in the biosynthesis of palladium nanoparticles in *Asterarcys* [47]. The involvement of polyols and amide groups in biosynthesis was demonstrated for *Chlorella vulgaris* [48].

A similar reaction between the crude extract of brown algae *Sargassum bovinum* and Pd^2+^ can occur when polyols and polysaccharides extracted from the algae are reduced and coated with octahedral PdNPs, thereby stabilizing them [49]. The proteins, alginates, or sulphated polysaccharides and polyols present in the *Saragassum cervicorne* extract have reduced Pd^2+^ ions to PdNPs and stabilized them by a capping process [50]. Brown alga *Padina boryana* facilitates the bioreduction of Pd^2+^ ions into Pd^0^. Subsequent growth of PdNPs is capped and stabilized by algal extract molecules adsorbed onto the particle surface. PdNP production began after the reduction of Pd^2+^ to Pd^0^ due to electrons released mainly from reducing sugars and polyphenols containing biomolecules of *P. boryana* extract [51]. The -OH functional groups from polyols, including terpenoids, tannins, saponins, etc., are also capable of forming complexes with PdNPs. In addition, tricosanoic acid and various esters were involved in surface capping and nanoparticle stabilization. The PdNPs had a pleomorphic shape and a size of 8.7 nm, and the value of the zeta potential (−28.7 ± 1.6 mV) indicated sufficient stability for effective use. This stability could be attributed to the efficient encapsulation of the PdNPs by the biomolecules present in the algal extract [52].

### 2.4. By Plant

Plants are regarded as the most promising avenue for the production of PdNPs, owing to their inherent renewability, vast biological diversity, favorable cost–benefit ratio in the synthesis process that avoids the need for toxic additives, and the long-standing recognition of their medicinal properties. These factors make plants a focal point in the advancement of this cutting-edge biotechnological domain. This type of PdNP synthesis is a simple process occurring at room or elevated temperature, where a palladium salt solution is mixed with a plant extract, resulting in nanoparticle synthesis.

The PdNPs’ synthesis can be achieved through various mechanisms, similar to those of other metal nanoparticles. These mechanisms are driven primarily by the unique conditions present where the synthesis occurs, with the “green factory” being the most critical factor (Figure 2).

Unlike bacterial synthesis, plant synthesis is more multifarious. Bacterial synthesis is largely enzymatic in nature, while the biomolecules capable of carrying out plant synthesis are more diverse in nature. Plant extracts have a complex composition, containing a spectrum of compounds belonging to different classes of organic matter. The substances included in natural extracts typically perform two major functions: acting as reducing agents and stabilizing nanoparticles. Plant synthesis is based on the main classes of organic compounds found in extracts. These include phenolic acids (gallic, chlorogenic, and ferulic), which possess strong reducing properties due to their hydroxyl (–OH) and acidic (–COOH) groups. COOH groups carry a negative charge and can effectively bind to partially positively charged surface metal atoms, thereby preventing their aggregation. Flavonoids (quercetin, rutin, and apigenin), thanks to their large number of OH groups, effectively reduce metal ions to atoms, which quickly aggregate to form nanoparticles. Furthermore, hydroxyl groups form hydrogen bonds with the surface of the nanoparticles, preventing their further aggregation. Essential oils (eugenol, carvacrol) contain phenolic fragments and free OH-groups in their structures, which can reduce metal ions. Terpenoids (geraniol, limonene, and carotenoids) contain numerous functional groups in their structures: hydroxyl, aldehyde, and keto groups, which participate in the reduction of metal ions from their precursors. Due to their N-containing heterocycles, alkaloids can form strong complexes with nanoparticles. Saponins can stabilize nanoparticles through the interaction of their hydrophobic and hydrophilic fragments and also form micelles around nanoparticles, improving their solubility. They can also induce the self-assembly of nanostructures. Polysaccharides (starch, chitosan, alginate, and cellulose) are environmentally friendly compounds that ensure the long-term stability of nanoparticles. Acting as capping agents, these compounds not only ensure the stability of PdNPs but can also impart remarkable therapeutic properties.

For example, in *Annona squamosa*, water-soluble compounds with hydroxyl functional groups were shown to play a role in both the reduction of palladium ions and the stabilization of PdNPs (Table 2) [53]. The phytochemicals in the *Euphorbia granulata* extract, particularly polyphenols, were responsible for both reducing the palladium salts and preventing the nanoparticles’ adhesion. The reduction mechanism lies in the fact that hydroxyl groups in plant phenols are oxidized during the reduction of Pd^2+^ to PdNPs, and polyphenols can be adsorbed on the surface of PdNPs, possibly by interacting through π-electron interactions in the absence of other strong ligating agents [54,55]. Bioreduction of Pd^2+^ ions to PdNPs was mainly due to the proteins and other phytochemicals present in the *Santalum album* extract [56]. The flavonoids in *Piper betle* extract could be adsorbed on the nanoparticle surface and thereby induce the reduction of Pd^2+^ to stable Pd^0^ nanoparticles by the interaction of carbonyl groups. In addition, terpenoids and proteins can participate in the PdNPs’ stabilization and capping [57]. *Coleus amboinicus* leaf extract contains carboxylic acids, which conduct the bio-reduction process that converts palladium into its nanoparticle form [58]. Organic compounds containing a carbonyl group, –OH groups (alcohol, phenolic), and a glycoside bond bind to PdNPs and form a coating layer [59]. Palladium ions can be reduced to Pd^0^ by *Eryngium caeruleum* flavonoids, and the synthesized PdNPs are coated and stabilized with functional groups, including free protein amino groups or flavonoid carbonyl groups [60]. The biosynthesis of PdNPs using *Chenopodium album* shoot extract is attributed to its constituent phytochemicals, such as saponins, steroids, polyphenols, and flavonoids [61]. The aqueous extract of *Sapindus mukorossi* contains a significant amount of saponins and flavonoids, providing a high number of hydroxyl groups involved in the Pd^2+^ reduction. In this case, bio-reduction of Pd^2+^ can occur through the oxidation of hydroxyl groups to form carbonyl groups [62,63]. Phytochemicals, such as flavonoids, mono- and sesquiterpenes, ursolic acid, and iridoid glycosides from the seed extract of *Lantana trifolia*, can act as reducing and stabilizing agents. Pd^2+^ ions can interact with –OH groups, oxidizing to the forms –CHO and –COOH, which leads to the reduction of Pd^2+^ to PdNPs under microwave irradiation, and, finally, the –COOH groups contribute to the PdNPs’ stability [64]. Phenolic compounds and their glycosides in *Origanum vulgare* extract play an important role in the redox reaction occurring during the reduction of Pd^2+^ to Pd^0^, while they are being oxidized to carboxyl groups [65]. Tannins from pomegranate extract could also act as reducing agents in the PdNPs’ synthesis [66,67]. The polyol components of the *Cinnamomum camphora* extract (flavones, terpenoids, and polysaccharides) were oxidized to aldehydes or ketones, while Pd^2+^ was reduced to elemental palladium [68]. The hydroxyl groups of starch and fatty acids from the broth bind to the surface of the nanoparticles, which stabilizes them and inhibits aggregation [69]. Bis-phthalate compounds containing a carboxylic functional group are responsible for the reduction of Pd^2+^ ions in *Moringa oleifera* [70]. Gallic acid has been used as a biologically active reducing agent in the synthesis of palladium nanoparticles, although other minor components cannot be excluded in *Garcinia pedunculata* [71]. Dauthal et al. suggested that the two hydroxyl groups in the benzene ring of gallic acid may be involved in the bioreduction of PdCl_2_. Whereas phenolic compounds can release electrons and are easily oxidized to the quinone form, PdCl_2_ first oxidizes gallic acid and forms an intermediate complex with palladium. Next, a palladium ion was formed with the concomitant oxidation of gallic acid into its quinone form. The Pd^2+^ ion was then reduced to Pd^0^ in the presence of free electrons or the resulting hydrogen, which were formed during the bio-reduction reaction. Finally, the collision of neighboring Pd_0_ atoms leads to the formation of PdNPs [72]. Pd^2+^ ions can form a chelated complex with polyphenolic compounds in the extract of the bark of *T. arjuna*. The chelated ortho-dihydroxy groups are then oxidized to quinones with the simultaneous reduction of Pd^2+^ to Pd^0^. The collision of Pd^0^ atoms leads to the formation of PdNPs, and the synthesized PdNPs are stabilized by polyphenolic compounds and quinones [73]. It was established in [74] that luteolin, kaempferol, and gentisic acid can act as excellent reducing agents. These reducing agents act as Lewis base ligands that can donate electron pairs to the central metal ion, and reduction occurs on that nanoparticle’s surface.

PdNPs stabilization is achieved by coating their surface with phytochemicals through binding interactions between metal atoms and oxygen-containing functional groups [75]. Aromatic compounds from the plant extract are also capable of acting as capping and stabilizing agents [76]. Steroids, saponins, tannins, alkaloids, and flavonoids present in the *Filicium decipiens* leaf extract may act as both reducing and stabilizing agents [77]. Flavonoids, tannins, and carbohydrates included in *Butea monosperma* leaf extract not only play a crucial role in the conversion of Pd^2+^ ions to Pd^0^, but also contribute to the PdNPs stabilization according to their strong binding ability [78]. The flavonoids demonstrate remarkable chelating characteristics, which enable them to coat palladium nanoparticles [78]. The water-soluble heterocyclic compounds, for example, flavones and proteins, can act as stabilizers for the PdNPs from poplar leaves [79,80]. The encapsulating layer on the nanoparticle surface could be formed by proteins from *Bauhinia variegata* [81]. The phenolic hydroxyl groups of flavones, terpenoids, and polysaccharides belonging to the phytomolecules of the *Rosa canina* fruit also have a strong ability to bind with PdNPs [82].

Phenolic compounds, such as eleutheroside B, vanillic acid, chlorogenic acid, catechin, epicatechin, ellagic acid, and taxifolin, and tannins and gallic acid, act as stabilizers for PdNPs. These substances, which are abundant in extracts from *Quercus* species, prevent the aggregation of synthesized palladium nanoparticles [83]. Moreover, polyphenols in plant extracts act as capping agents, conferring a negative charge to the nanoparticle surface. This charge prevents aggregation and is essential for controlling the resulting nanoparticle morphology [74]. High negative values of the zeta potential also indicate the presence of a high electric charge on the surface of *Chrysophyllum cainito* and *Cassia absus*-mediated PdNPs, making them more stable due to capping molecules on the surface [84,85].

The described biocompounds involved in the synthesis and stabilization provide clear benefits, enhancing the characteristics of the resulting PdNPs [86]. For example, flavonoids enable the production of bioactive and biocompatible nanoparticles and improve interactions with biological structures, increasing the selectivity of biomedical action.

All of the above demonstrate the wide diversity of capping molecules from plant extracts. They share medicinal properties with both bacterial and fungal biosynthesis, which can enhance the biomedical effect of the palladium nanoparticles themselves. The interaction of nanoparticles with biomolecules underlies the development of nanotherapeutic systems, vaccines, diagnostic platforms, and targeted delivery systems. Control of capping agents and the nanoparticle surface allows for increased specificity, decreased toxicity, and improved pharmacokinetic properties. Moreover, the ever-expanding range of plant extracts used for the synthesis of PdNPs may broaden our understanding of both the biosynthetic mechanism and potentially promising biological compounds for biomedical applications.

**Table 2:** PdNPs from plants.

Source	Part of Plant	Size, nm	Shape	Ref.
*Annona squamosa*	peel	80 ± 5	spherical	[53]
*Euphorbia granulate*	leaves	25–35	spherical	[54]
*Allium fistulosum*, *Basella alba*, *Tabernaemontana divaricate*	leaves	20–50	spherical	[55]
*Santalum album*	leaves	10–40	spherical	[56]
*Piper betle*	leaves	4	spherical	[57]
*Coleus amboinicus*	leaves	20	spherical	[58]
*Butea monosperma*	flower	3–8	spherical	[59]
*Eryngium caeruleum*	leaves	20.5	spherical	[60]
*Chenopodium album*	shoot	70–90	spherical	[61]
*Sapindus mukorossi*	seeds	3–5	spherical	[62]
*Curcuma longa*	root	50–150	spherical	[63]
*Lantana trifolia*	seeds	5–15	spherical	[64]
*Origanum vulgare*	aerial parts	2–20	spherical	[65]
*Matricaria recutita*	flower	3.66	spherical	[66]
*Punica granatum*	peel	20–24	spherical	[67]
*Cinnamomum camphora*	leaves	3.2–6	spherical	[68]
*Pistacia atlantica*	fruit	10	spherical	[69]
*Moringa oleifera*	flower	10–50	spherical	[70]
*Garcinia pedunculata*	leaves	2–4	spherical	[71]
*Delonix regia*	leaves	2–4	spherical	[72]
*Terminalia arjuna*	bark	4–16	spherical	[73]
*Solanum nigrum*	leaves	21.55	spherical	[74]
*Gymnema sylvestre*	leaves	10–20	spherical	[75]
*Lagerstroemia speciosa*	leaves	136.5	-	[76]
*Filicium decipiens*	leaves	2–22	spherical	[77]
*Butea monosperma*	leaves	3–25	spherical	[78]
*Parthenium hysterophorus*	leaves	~4 nm	spherical	[79]
*Poplar*	leaves	2.2–6.8	spherical	[80]
*Bauhinia variegata*	leaves	2–9	irregular	[81]
*Rosa canina*	fruit	~10	spherical	[82]
*Quercus dalechampii*, *Quercus petraea*, *Quercus frainetto*	bark	63.9 ± 8.3 63.1 ± 8.2 48.0 ± 6.2	-	[83]
*Cassia absus*	seeds	~12	spherical	[84]
*Peganum harmala*	Seeds	22.5	spherical	[87]
*Platycladus orientalis*	leaves	~8	spherical	[88]

While the size of plant-derived PdNPs can vary, they are most commonly spherical in shape (Table 2). Smaller-diameter nanoparticles are of particular interest for future biomedical applications due to their enhanced ability to penetrate cells and their higher biological activity. It has been reported that 100 nm nanoparticles exhibited a 2.5-fold greater uptake compared to 1 µm diameter particles and a 6-fold greater uptake than 10 µm particles. As particle size gets smaller, their surface area to volume ratio gets larger. Moreover, different studies show that spherical particles are good candidates for drug delivery because they can be internalized with high likelihood and faster than rod-shaped NPs [89]. Based on these characteristics, PdNPs appear to be attractive drug candidates. The reason why some plant extracts produce palladium nanoparticles with a narrow size distribution, while others produce a broad one, is unknown. It is possible that the broad size distribution is due to the presence of several biomolecules that can act in parallel as reducing and stabilizing agents, each leading to the formation of palladium nanoparticles of different sizes [4].

### 2.5. By Other Sources

Palladium nanoparticles can also be synthesized using the plant compounds themselves. These nanoparticles, with their predetermined properties and stabilized by capping molecules, hold considerable promise for future applications in the field of therapeutics and medicine. For instance, gum ghatti (*Anogeissus latifolia*) is an arabinogalactan type of natural biopolymer, which contains sugars such as arabinose, galactose, mannose, xylose, and glucuronic acid. The hydroxyl and carboxylic groups of the gum complex the palladium ions, which can be further reduced to elemental palladium by the reducing aldehyde groups. Then, these nanoparticles are possibly capped and stabilized by the gum proteins and polysaccharides [90]. As a result, spherical PdNPs with a size of 2.3 to 7.5 nm were produced [90]. Gum acacia is a highly branched, neutral or slightly acidic arabinogalactan polysaccharide obtained naturally from the stems and branches of the *Acacia senegal* tree and has been shown to be an effective capping agent because of the well-established chemical bonding between the –OH and –COOH functional groups and the surface of the metallic nanoparticles [91]. Hydroxyl groups on the surface of guar gum are believed to be responsible for the Pd^2+^ reduction. The carboxylic and hydroxyl groups of guar gum form a complex with the Pd^2+^ ion and finally reduce it to Pd^0^. Guar gum also contains starchy compounds that act as a stabilizing agent and help in the prevention of PdNPs’ aggregation in water [92]. The *Pistacia atlantica* kurdica gum contains phenolic and triterpenoid groups that can form complexes with Pd^2+^ ions in solution, thereby altering the palladium oxidation state.

The triterpenoids and phenolic compounds present in the gum can play a crucial role in reducing Pd^2+^, while the starch compound found in this gum acts as a stabilizing agent, preventing the aggregation of synthesized PdNPs in aqueous solutions [93]. Pd^2+^ will undergo complexation with hydroxyl and carbonyl functions of various phytochemicals (polysaccharides, essential oils, proteins, and terpenoids) present in Frankincense resin (a naturally occurring gum resin obtained as an exudate from the bark of the *Boswellia sacra* tree). Due to the strong antioxidant nature and high reducing capacity, these hydroxyl and carbonyl functions will reduce Pd^2+^ to Pd^0^ atoms and undergo oxidation to carboxyl groups. Pd atoms serve as nucleation sites for the growth of PdNPs. Finally, the size control and stabilization of PdNPs is achieved by the surface coating of these phytochemicals through binding interactions between metal atoms and oxygen-containing functional groups [94]. Propolis extract contains biomolecules that reduce palladium ions to PdNPs, including terpenoids, flavonoids, and phenolic substances. In addition, flavonoids also act as a capping agent in the PdNP production [95].

Quercetin diphosphate, a naturally derived flavonoid, was used for the production of PdNPs. This compound has both C- and P-bound hydroxyl groups. These groups can freely chelate with the Pd^2+^ ions. Moreover, Pd^2+^ ions have a high redox potential, contributing to the stability of the final stage of nanoparticle synthesis [96]. Polysaccharides from *Strychnos potatorum* seeds play a dual role in the PdNP synthesis process as a capping and reducing agent [97]. *Ginkgo biloba* leaf polysaccharide, a water-soluble biomacromolecule with high antioxidant activity, was used as a reducing agent and stabilizer in the synthesis of PdNPs [98].

PdNP synthesis was carried out using tannic acid. The polyphenolic group present in the tannic acid is responsible for the reduction of the Pd^2+^ ion. During the reduction process, the –COOH present in the tannic acid becomes COO–. This COO—along with the rest of the polymer—can act as a surfactant to attach to the PdNPs surface, and it stabilizes PdNPs [99].

Owing to its polycationic nature and conformation in solution, chitosan is a good option for both stabilizing palladium nanoparticles and regulating their size. The size of the nanoparticles can be adjusted between 25 and 150 nm, making them suitable for a wide range of biotechnological applications [100].

This form of synthesis allows for more precise control over the properties of palladium nanoparticles. For example, flavonoids can act as antioxidants and exert hepatoprotective and antibacterial effects, while terpenoids demonstrate anticancer potential. Furthermore, studies of pure compounds are very helpful in determining the mechanism of nanoparticle formation and stabilization using crude extracts.

## 3. Potential Biomedical Application of Green PdNPs

**Figure 3:**
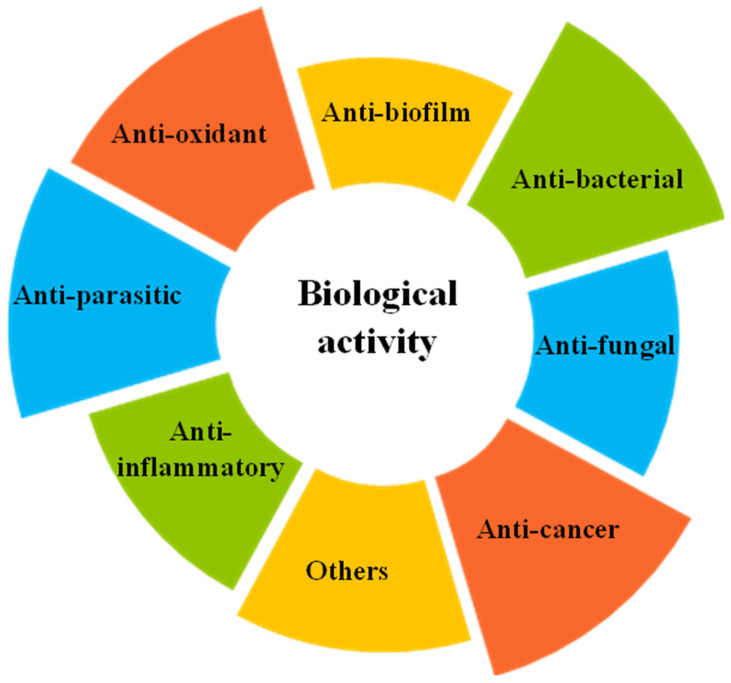
Potential biomedical application of green PdNPs.

Despite significant advances in nanotechnology, the introduction of palladium nanoparticles into clinical practice remains a long way off. Successful implementation requires a comprehensive understanding of the physicochemical properties, in vitro and in vivo effects, toxicity, biodistribution, pharmacokinetics, and pharmacodynamics of palladium nanoparticles. Nevertheless, considerable progress has already been made toward this goal. This includes the characterization of several potentially beneficial properties of PdNPs (Figure 3). A deeper understanding of these traits could be key to their eventual clinical use.

### 3.1. Antibacterial Activity

Antibiotic resistance of pathogenic microorganisms is a major challenge for modern medicine. The pursuit of novel antimicrobial agents that are not only effective but also devoid of adverse effects on human health remains a critical objective, with the solution holding the promise of significantly alleviating the burden on healthcare systems. In comparison to conventional antibiotic medications, metal nanoparticles have exhibited exceptional efficacy against pathogenic microorganisms in a multitude of studies. In order to select the most effective drug, it is also essential to comprehend the mechanisms that underlie these potential treatments.

The antibacterial activity of PdNPs is not fully understood, but it is assumed to follow the general principles of other metal nanoparticle activity. Two main pathways can be identified: the first involves the disruption of the cell membrane, leading to the leakage of cellular contents and binding to proteins, causing their denaturation and ultimately cell death (1). The second presupposes penetration into the cell and damage to intracellular structures such as ribosomes and DNA, inactivation of the respiratory chain, and the reactive oxygen species generation (ROS) (2) [101]. Furthermore, there are various factors that impact the antimicrobial activities of palladium nanoparticles, such as particle shape, size, morphology, and surface charge.

One of the possible mechanisms for the PdNPs’ antibacterial activity is their ability to adhere to the bacterial cell surface. This adhesion is due to the electrostatic interaction between positively charged palladium ions and the negatively charged cell membranes. This allows the nanoparticles to penetrate the cell membrane, causing structural changes that lead to bacterial death. The antibacterial activity of the PdNPs against bacteria might have been due to the interaction of Pd^2+^ with the phosphate and carboxylate groups of lipopolysaccharides (LPS) present in the cell wall, based on the electrostatic force of attraction, alteration of the cell membrane permeability, and enhancement of ROS production, causing oxidative stress-mediated cell death [102]. For example, *E. coli* and *S. aureus* cells treated with *Orthosiphon stamineus*-PdNPs showed abnormal structures, such as membrane rupture and disintegration, and loss of contact between neighboring cells, leading to cell clumping and aggregation. Additionally, PdNPs were observed on the surface of the bacterial cell wall, confirming the interaction of PdNPs with the bacterial cell membrane, contributing to its damage and the permeability of nanoparticles into the cell, and provoking ROS-mediated cell death [102].

A critical aspect of the impact on bacterial cells is the configuration of the cellular wall, which varies between Gram-positive and Gram-negative bacterial species. The cell wall of Gram-negative microorganisms is composed of a thin layer of peptidoglycan, which consists of linear polysaccharide chains linked together with short peptides. This arrangement forms thinner structures that allow for the easy passage of nanoparticles. In contrast, the cellular wall of Gram-positive microbes features a thicker layer of peptidoglycans. Gram-negative microorganisms (*Pseudomonas aeruginosa*, *Klebsiella pneumonia*) were more vulnerable to PdNPs than Gram-positive microorganisms (*B. subtilis*, *Enterococcus faecalis*) [103]. PdNPs from Urtica extract exhibited higher antibacterial activity against Gram-negative than Gram-positive bacteria [104]. At the same time, palladium nanoparticles synthesized by lemon peels proved to be comparably effective against both Gram-negative and Gram-positive bacteria [105]. The minimum inhibitory concentration of PdNPs at 100 µg/mL was identical for both Gram-positive (*Staphylococcus aureus*) and Gram-negative bacteria (*E. coli* and *K. pneumoniae*) [43]. *Couroupita guianensis*-mediated PdNPs have shown remarkable antibacterial activity against both Gram-positive (*S. aureus*, *R. rhodochorous*, *B. cereus*, *M. luteus*) and Gram-negative (*E. coli*, *Proteus mirabilis*, *Ps. aeruginosa*, *Salmonella typhi*, *Vibrio cholerae*, *K. pneumonia*) bacterial pathogens at 25 μg/mL dosage [106]. The *Salmalia malabarica* gum-mediated PdNP’s antibacterial activity was observed against Gram-positive (*B. subtilis* and *B. cereus*) and Gram-negative *(E. coli*) [107]. *Daucus carota*-PdNPs’ exposure to Gram-negative bacteria resulted in a maximum effect against *V. cholerae* [108].

Studying the interactions of nanoparticles with microorganisms is crucial for a comprehensive understanding of nanomaterial toxicity and their potential as antibacterial agents. It is known that the behavior of NPs is determined by the size and composition of the particles, although the impact of small differences in size on biological cells remains poorly understood. A significant difference in the size dependence of antimicrobial activity, which differed based on the microorganism tested, was early observed for chemically produced PdNPs [109]. The smaller PdNPs have better antibacterial properties in comparison with the bigger ones. Similar data were obtained for biosynthesized PdNPs. The smaller NPs, which have a larger surface area, show higher antibacterial activity at low concentrations, whereas bigger NPs show antibacterial activity only at high concentrations [24]. Their antibacterial effect may be due to a larger specific surface area (compared to larger nanoparticles) and, therefore, a larger area available for interaction with microbial cells and for a more rapid release of metal ions [110].

A size-dependent correlation with antimicrobial activity has also been demonstrated: nanoparticle size plays an important role in their antimicrobial activity against both Gram-positive and Gram-negative bacteria. For example, larger nanoparticles interact only with the cell membrane, while smaller nanoparticles are potentially easier to penetrate the cell membrane by endocytosis [111]. It was postulated that the primary mechanism involved in the bacterial cell wall disruption was the interaction between palladium ions and sulfur-containing proteins present within the cell wall [95].

PdNPs from small brown algae, *Padina boryana*, with a large surface area and a biologically active coating material, effectively suppressed the growth of pathogenic microorganisms [51]. The interaction of bacterial cells with PdNPs coated with biomolecules from algae extract can occur through attachment to the peptidoglycan layer, due to the bond between free amino groups, hydroxyl, carbonyl, epoxy, or ester groups in capping biomolecules. This binding also promotes the penetration of PdNPs into the periplasmic space and the creation of new pores in the cell membrane through physical interaction with phospholipids and lipid peroxidation of the membrane, leading to a change in membrane permeability and inactivation of cellular enzymes. Indirectly, the encounter and binding of –SH groups of proteins with PdNPs can trigger a modification of the PO_4_^−^ efflux system leading to cell membrane exfoliation from cytoplasm, intracellular oxidative stress, dysfunction of the DNA replication system, and leakage of cell content [51]. *Curcuma longa*-mediated PdNPs triggered damage to the cell membrane, causing leakage of protein, minerals, and genetic material through the formed lesions, which led to cell death. In addition, the interaction between PdNPs and pathogenic bacteria can result in stress on the cell wall, provoking lactate dehydrogenase production and possible cell damage [65].

Proteins, minerals, and genetic material are released through the damaged cell membrane, inducing cell death. The amount of protein released from cells increases with increasing concentration and period of contact with *Candida krusei*-PdNPs [36]. Once the intracellular content is released into the cell suspension, it results in stress in the cell wall, thereby producing more lactate dehydrogenase and leading to cell damage with prolonged exposure time. The synthesis of alkaline phosphatase in the periplasmic region also substantially rose in *S. aureus* and *S. mutans* compared to *E. coli*, possibly due to the oxidative stress caused by nanoparticles on bacteria and their ability to overcome phosphate deficiency [36].

When PdNPs interact with the membrane of a microbial cell, metal ions tend to penetrate into the cell, thus causing oxidative stress inside the bacterial cell and ultimately its death. It was found that the production of intracellular ROS depends on the *Eryngium caeruleum*-PdNPs dose, and the bacterial cell interaction with nanoparticles leads to the penetration of metal ions into the cell, resulting in inhibition of the respiratory enzyme, which otherwise would destroy intracellular ROS, thereby accelerating the ROS production. Subsequently, these ROS damage various cellular components of bacteria, including DNA, cell membranes, and enzymes, causing cell death [60,111]. In [112], nanoparticles from *Andrographis paniculata* disrupt the integrity of the cell wall and cause oxidative stress through the ROS generation. ROS generation is considered to be the main mechanism of PdNPs’ action on cells. They interact with the bacterial cell membrane and easily penetrate into the cytoplasm. Inside the cytoplasm, the metals release ions that generate ROS, which directly affects DNA, inactivates enzymes, damages membranes, and causes all organelles to escape, resulting in cell death [113].

It is proposed that PdNPs penetrate into the bacterial cell and alter the normal synthesis of nucleic acids, which leads to cell death or at least DNA damage [95]. Small *Rosmarinus officinalis* metal nanoparticles, specifically spherical ones, are interacting with bacterial DNA, resulting in cross-links and distortion of the spiral structure [114]. On the other hand, the ribosome subunit stops binding to tRNA [75].

**Figure 4:**
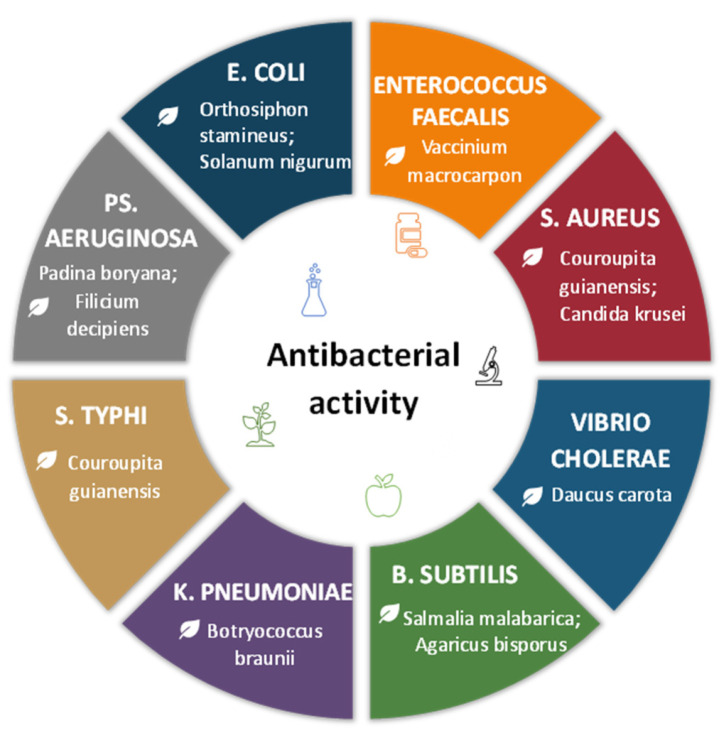
The antibacterial activity of some green PdNPs. Sources for the biosynthesis are indicated by a 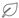.

Additionally, the bactericidal action may be influenced by phytochemicals found in the extract acting as a capping agent on the surface of PdNPs [103,114]. Thus, the antibacterial effect may be synergistic due to the nanoparticles and these capping agents. For instance, the antimicrobial activity of the neem gum-mediated PdNPs might be by virtue of the presence of linalool, methyl esters of ricinoleic acid, linoleic acid, oleic acid, and ethyl esters of stearic acid [102]. *Solanum nigrum*-PdNPs had an impact on Gram-negative *E. coli* by binding capping polyphenols to a microbial protein. It changes the membrane potential and reduces the synthase activities of adenosine triphosphate, slowing down the metabolism [75]. The high antibacterial PdNP activity may be related to the influential role of a heterogeneous group of phenolic compounds in *Agaricus bisporus*, which served as potential biologically active molecules capping PdNPs [41]. The *Filicium decipiens* leaf extract phytochemicals present as capping agents on the PdNPs surface may have influenced the bactericidal actions against Gram-negative bacteria [77]. The antibacterial activity of some green PdNPs is presented in Figure 4.

Available literature data support the active role of capping agents in the antibacterial activity of PdNPs. Possessing their own antibacterial potential, they can enhance the effect. However, identifying phytochemicals with medicinal activity is a complex and challenging process, but such studies are necessary to elucidate their precise role in antibacterial activity.

### 3.2. Antibiofilm Activity

Biofilms, which are microbial communities comprising one or more species of bacteria surrounded by an extracellular matrix primarily composed of polysaccharides, nucleic acids, and proteins, exhibit remarkable resistance to antimicrobial agents. Recent research has revealed that PdNPs exhibit antibiofilm properties. Applied at a concentration of 125 µg/mL, PdNPs produced by the extract of *Padina boryana* completely prevented biofilm formation by strains of *P. mirabilis*, *S. aureus*, *Acinetobacter pittii*, *Ps. aeruginosa*, *E. fergusonii*, and *Aeromonas enteropelogenes* [52]. The PdNPs’ effectiveness against *S. aureus* and *Ps. aeruginosa* biofilms was found, causing significant damage, including the morphology changes and PdNP accumulation on the bacterial cell wall and biofilm [115]. Under the influence of PdNPs on a new clinical isolate of multidrug-resistant (MDR) *Cronobacter sakazakii*, the biofilm biomass significantly decreased. The stress exerted by nanoparticles on microbial cells leads to a decrease in their adhesion to the substrate. The *Orthosiphon stamineus*-PdNPs triggered a dose-dependent inhibition of biofilm formation by methicillin-resistant *Staphylococcus aureus* (MSSA), and a disrupted biofilm architecture with a minimal number of microcolonies was revealed. This is apparently associated with the decrease in adhesion of microorganisms to the growth surface [102]. The destruction of biofilms by *Andrographis paniculata*-PdNPs can be explained by the nanoparticles’ ability to interact with the biofilm matrix and destabilize it, leading to the release of cellular contents and weakening of the structure. These nanoparticles not only hindered the new biofilm formation but also contributed to the destruction of pre-existing ones, underscoring their potential as an effective treatment for oral infections associated with biofilms [114].

### 3.3. Antifungal Activity

Fungal infections, commonly referred to as mycoses, constitute a group of human illnesses that affect the skin, nails, mucous membranes, scalp, and internal organs. These infections are caused by diverse types of fungi and may be challenging to manage due to the emergence of resistance to current therapies. It was demonstrated that palladium nanoparticles can effectively inhibit the growth of these harmful fungi, making them a promising approach for combating fungal infections. Nevertheless, further research is required to fully comprehend their efficacy and safety. According to studies, chemically produced PdNPs caused an increase in ROS levels, cell wall damage, and cellular morphology changes in *Candida albicans* and *Aspergillus niger* [116]. The green PdNPs will cause an increase in hydroxyl radicals, can bind to phosphate groups of DNA or other macromolecules on the outside of the cell, play a role as an enzyme inhibitor, involve interactions with cellular components, and trigger damage to the fungal cell. The mechanism can also be based on the metal nanoparticle penetration into the cell membrane. It increased effects on the respiratory chain and the termination of cellular division, ultimately leading to cell death [114]. While nanoparticle size was a significant factor in the antifungal activity against *Colletotrichum gloeosporioides*, it was not the sole determinant of efficacy against *Fusarium oxysporum* [96]. In other studies, PdNPs synthesized using *Rosa damascena* flower extract demonstrated antifungal effects against *Aspergillus niger*, *A. flavus*, and *Candida albicans* [117]. Similarly, PdNPs derived from turmeric extract exhibited dose-dependent antifungal activity against *Candida* spp., with results comparable to the standard drug fluconazole [63]. Furthermore, colloidal PdNPs were found to inhibit the growth of *A. niger*. As expected, a rise in PdNP concentration correlates with an increase in the inhibition zone diameter [57]. PdNPs synthesized from different *Quercus* extracts demonstrated notable efficacy against *Candida albicans*, *Candida krusei*, and *Candida auris*. The enhanced antifungal effect observed against *C. krusei* may be attributed to a synergistic interaction between the palladium nanoparticles and the bioactive constituents of the *Quercus* extracts. It is suggested that PdNPs compromise the integrity of the fungal cell wall, thereby improving the uptake and efficacy of the extract’s active components, ultimately resulting in more pronounced growth inhibition [83].

### 3.4. Antioxidant Activity

ROS, including superoxide, NO, –OH radicals, and hydrogen peroxide, are naturally occurring mutagenic, highly reactive radicals. These radicals can induce oxidative damage to cellular proteins and other macromolecules, contributing to the pathogenesis of various diseases, including inflammation, atherosclerosis, aging, cancer, and neurodegenerative disorders. However, cells possess antioxidant mechanisms that protect against damage by scavenging radicals, binding metal ions, and inhibiting radical formation. PdNPs may be useful for this purpose. One of the most popular and fast methods for assessing antioxidant activity is using DPPH (1,1-diphenyl-2-picrylhydrazyl radical), which is a standard tool for measuring the ability of compounds to neutralize free radicals. In many studies, PdNPs were found to have an antioxidant effect in a dose-dependent manner—increasing the concentration of PdNPs enhances antioxidant activity [56,63,104]. Such data were obtained, for example, for *Curcuma longa*-mediated [56], *Azadirachta indica*-mediated [118], and *Cassia absus*-mediated PdNPs [85]. It was detected that the antioxidant activity of smaller nanoparticles is higher than that of large ones, because their size strongly affects the absorption activity of NPs [67,97]. In addition, capping agents with their own antioxidant activity enhance the effect and tend to absorb free radicals [59,119]. For example, *Saururus chinensis* with a high content of phenolic compounds, *Coleus aromaticus*, and *Myristica fragrans* essential oils have an outstanding antioxidant potential because their composition mainly includes terpenoids and phenolic derivatives, creating a synergistic effect of the nanoparticles and their phytocomponents [119,120]. PdNPs from *Coleus aromaticus* and *Myristica fragrans* showed exceptional antiradical action against the –OH radical, while the maximum percentage of inhibition was achieved at higher concentrations of the antioxidant [120]. The antioxidant activity of PdNP-*Salvia hispanica* may also be attributed to the presence of flavonoids, alkaloids, and phenolic compounds, which can absorb free radicals and prevent their formation [114]. Polysaccharides (especially alpha and beta glucans) and phenolic acids (catechin, gallic acid, rutin, caffeic acid, and pyrogallol) capped on the *Agaricus bisporus*-PdNPs have a pivotal role in enhancing the antioxidant effect against the DPPH radical [41]. Certain scholars even postulate that the antioxidative capacity of PdNPs is more closely linked to the content of phenolic compounds in the extract rather than to the inherent PdNP properties [83].

### 3.5. Anticancer Activity

At the beginning of the 21st century, cancer became a significant public health concern, as the number of cases detected worldwide has been steadily increasing. Modern drug treatments often come with a range of side effects, which can significantly decrease patients’ quality of life. Therefore, the search for new medications with effective anticancer properties and good tolerance remains a top priority in the fight against cancer.

**Figure 5:**
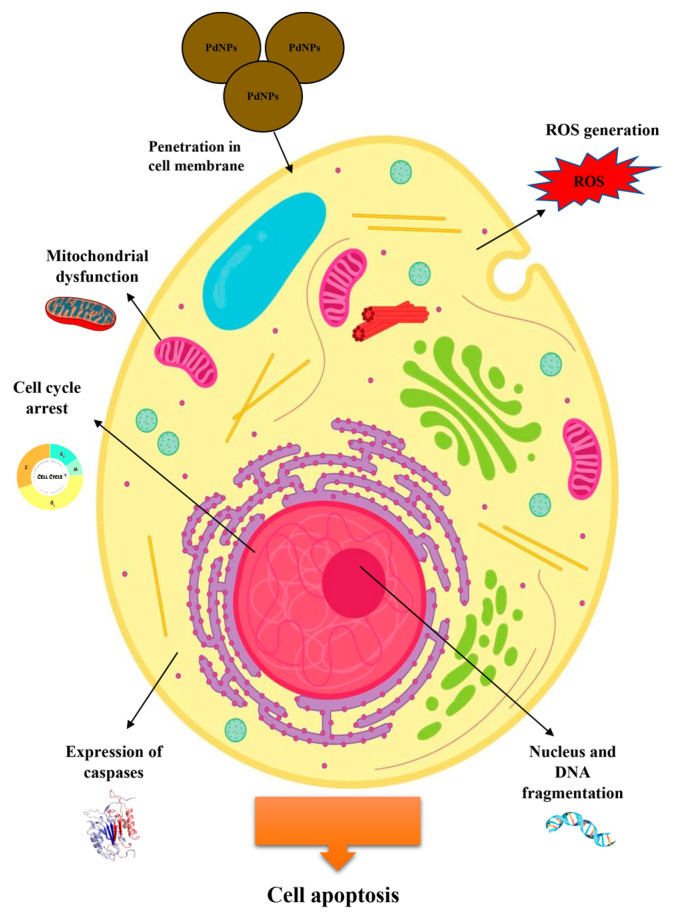
The proposed mechanism of PdNP anticancer activity.

The available literature data suggest PdNPs exhibit anticancer properties against various types of cancer cells. The proposed mechanism underlying the anticancer effect of PdNPs proposed several fundamental principles (Figure 5): ROS generation (1), disruption of mitochondrial membrane potential (MMP) (2), activation of caspases responsible for apoptotic cell death (3), DNA fragmentation (4), induction of autophagy and subsequent autophagic cell death (5), cytotoxicity (6), and cell death [121].

In vitro. *Moringa oleifera*-mediated PdNPs may possess anti-proliferative activity in A549 cells [70]. PdNPs caused significant cytotoxicity to A549 cells and did not induce toxicity in normal healthy peripheral lymphocytes [63]. PdNPs from *Cannabis sativa* leaf extract had similar cytotoxic effects on A549 lung cell lines [122].

PdNPs showed significant inhibition of human breast cancer MCF-7 cell proliferation in a dose-dependent manner. MCF-7 cells treated with PdNPs underwent shape changes, such as cell size reduction and rounding to varying degrees [63]. PdNPs synthesized by *Saussurea costus* were also effective against cancer cells MCF-7, almost as much as HCT-116 (colon cancer cell line) and HepG-2 (human liver cancer cell line) [123]. Biogenic PdNPs from *Vaccinium macrocarpon* significantly suppressed the MCF-7 cancer cell proliferation, exceeding the effectiveness of the conventional drug doxorubicin [103]. The MTT assay demonstrated that PdNPs from turmeric extract have an excellent cytotoxic effect against HCT116 [124]. They can induce apoptosis in colorectal cancer cells in a dose-dependent manner with a lower CC50 (78 μL).

Defects in the normal cell cycle arrest response to DNA damage may lead to the development of cancer, and the induction of cell cycle arrest is an important element of the antitumor effect of many chemotherapeutic agents widely used in clinical practice. Therefore, control of the cell cycle is the main event in the process of cell division, making its specific checkpoints—particularly the G2/M transition—critical targets for anticancer therapy. The prevention of mitosis (M-phase) stimulates the induction of apoptotic pathways. It was found that *Camellia sinensis*-PdNPs induced G2/M phase arrest in WEHI-3B cell cells. Leukemic cells in the spleen were reduced by *C. sinensis*-PdNPs with an increase in Bax/Bcl-2, cytochrome C protein, and mRNA levels, indicating the activation of the mitochondrial apoptotic pathway [125]. In addition, capping agents, flavonoids, which can control the metabolic activity of cancer cells, may also be important. The flavonoid antitumor effects are manifested in oxidative degradation, inhibition of proliferation, inactivation of carcinogens, stimulation of differentiation, induction of cell cycle arrest and apoptosis, disruption of tumor angiogenesis, and suppression of metastasis. Saudi propolis plays a special role as a capping agent in anticancer activity on MCF-7 cells [95]. *Bauhinia variegata*-PdNPs exhibited potent anti-proliferative efficacy against MCF-7 cells in a dose-dependent manner; moreover, they are more effective than the standard drug doxorubicin [81]. It is suggested that *Bauhinia variegata* in nanoparticles plays an important role in enhancing the cytotoxic effect. The antitumor activity of PdNPS from *Punica granatum* against A549 and MCF7 cells in vitro showed a decrease in viability by 43% and 32%, respectively. Anticancer activity has also been confirmed in vivo [126].

PdNPs from ginger extract demonstrated a significant ability to induce apoptosis in HCT116 [127]. It inhibited cell division in the G1 and S phases, resulting in a cell frequency of 38.6 and 34.9% in each phase, respectively. REG4 is an anti-apoptotic, prognostic diagnostic factor in colorectal cancer (CRC) and an invasive factor in CRC cell lines, associated with liver metastasis. At the same time, CAT (catalase) activity is reduced in cancer. PdNPs have shown promising therapeutic results by significantly reducing the activity of the REG4 gene, a CRC marker, and upregulating the CAT antioxidant gene [127]. Similar results were demonstrated for PdNPs functionalized with quercetin: MTT assays showed significant inhibition of HCT-15 colorectal cancer cell proliferation, and activation of apoptosis in cancer cells [128].

*Gloriosa superba*-PdNPs showed powerful antitumor activity against MCF-7 cells (human breast adenocarcinoma). The cell death mechanism is the apoptosis induction characterized by the release of phosphatidylserine, membrane disintegration, and the formation of vesicles with chromosome condensation [129]. The mechanism of antitumor activity of *Barleria prionitis*-PdNPs was also found to be the externalization of phosphatidylserine and increased membrane permeability, which are considered to be the hallmarks of apoptosis [130].

PdNPs synthesized using rosemary and ginseng extracts had an effect on colon cancer cell lines (SW480 and LS180). They induced cell death and influenced the expression of autophagy-related genes [131].

Human ovarian cancer cells A2780 treated with *Evolvulus alsinoides*-PdNPs died: increased LDH leakage and ROS generation, impaired mitochondrial membrane potential (MMP), induction of autophagy and death of autophagic cells, and increased caspase-3 activity and DNA fragmentation were found as hallmarks of apoptosis [121]. PdNPs trigger cellular toxicity via ROS generation, which results in autophagy and eventually leads to autophagic cell death. Moreover, smaller-sized PdNPs induce higher levels of ROS than larger particles do. Since the mitochondria may produce massive amounts of ROS that can modulate autophagic processes, it is hypothesized that the excessive generation of ROS induced by PdNPs could trigger autophagy and eventually lead to autophagic cell death. Cells treated with PdNPs exhibit internalization of PdNPs into the cytoplasm, which induces the formation of numerous autophagosomes. Interestingly, PdNPs not only induced autophagosomes but were also localized in autophagosomes and autolysosomes. In addition, activation of autophagy was accompanied by an increase in ROS levels in cells exposed to PdNPs [122].

Anticancer activity against HeLa cells is due to apoptosis by *Dioscorea bulbifera*-PdNPs [132]. In another report, the PdNPs prepared from *Syzygium aromaticum* activate cytochrome c and caspase 3, downregulate Bcl-2 and Bcl-xL, and induce apoptosis-mediated HeLa cell death [133]. *Padina boryana*-mediated PdNPs induced the expression of apoptosis marker genes: p53, caspase-3, bax, and caspase-9 [48]. Increased expression of p53 mRNA transcripts indicates multiple targets for PdNPs in the MCF-7 cell line, including oxidative stress, mitochondrial dysfunction, cell cycle disruption, and apoptosis. Similarly, bax is a well-known inducer of apoptosis. Increased expression of two major caspases (caspase-9 and caspase-3) highlights the fragmentation of nuclear material and indicates the role of mitochondria in p53 apoptosis. Moreover, higher expression of p53 enhances transcription of bax, caspase-9, and caspase-3 as pro-apoptotic genes [48].

Using the MTT assay, distinct morphological features of the PK13 cell line (kidney cancer cell lines) have been shown, such as cell shrinkage with thickened cytoplasm and fragmented nuclei, confirming the induction of apoptosis under the influence of PdNPs from *A. bisporus* [41]. The significant cytotoxic effect of PdNPs may be due to the small particle size (15–18 nm), facilitating the PdNPs’ penetration through the cell membrane to induce cytotoxicity.

An extremely interesting study investigated the anticancer properties of PdNPs functionalized with resveratrol, a biochemical compound abundant in the skins of black grapes [134]. It is well known that the migration of cancer cells plays a crucial role in distant metastases, and therapies that can prevent this migration may become new weapons in the fight against cancer by inhibiting cell proliferation. Thus, in vitro, effective inhibition of PC-3 prostate cancer cell migration under the influence of Res-PdNPs was shown, demonstrating the ability of this nanomedical agent to stop cytoskeletal activity, therefore limiting intercellular interactions with neighboring cells. Nuclear transcription factor (NF-kB) is known to increase cell survival, tumor invasion, metastasis, and chemoresistance in human cancer progression. It was shown that the ability of Res-PdNPs to target the NF-κB signaling pathway and their efficacy in the induction of polarization of macrophages to the anti-tumor phenotype by inhibiting NF-κB phosphorylation, with consequent promotion of anti-tumor cytokines, such as interleukin 12 [134].

In vivo. Res-PdNPs can effectively slow down tumor growth in severely compromised immunodeficient (SCID) mice with prostate cancer (PC-3 cells) [134]. Analysis of CD31 tissue biomarkers showed that Res-PdNPs inhibit angiogenesis in tumor tissue. The authors hypothesize that the nature of the binding interaction of resveratrol on the surface of the PdNPs is optimal for the selective and effective delivery of Res and PdNPs to tumor cells/tumor tissue [134].

As can be seen, nanoparticle size is important for the future use of nanoparticles in anticancer therapy. For example, nanoparticles having a diameter of less than 200 nm may take advantage of the EPR effect for improved drug accumulation in tumors since tumor blood arteries have fenestrations ranging from 0.2 to 1.2 μm [135]. Most nanoparticles must be less than 200 nm in size to fully utilize their potential as anticancer drugs. Therefore, nanoparticle distribution and size must be precisely controlled throughout all biosynthetic steps. Functionalization of palladium nanoparticles with various capping agents can not only enhance their anticancer effects but also improve their biocompatibility. Furthermore, biocompounds with tailored properties can be used to modify PdNPs for targeted drug delivery, increased bioavailability, and controlled drug release from a single dose.

### 3.6. Other Activities

Beyond the biological effects already mentioned, palladium nanoparticles have been found to exhibit other notable properties. For instance, anti-inflammatory and analgesic activities were observed in a rat model using PdNPs derived from *Rosa damascena* flower extract [136].

Malaria is one of the most common and dangerous diseases in Africa, transmitted to humans through the bites of female mosquitoes belonging to the genus *Anopheles* (also known as “malaria mosquitoes”). This disease is caused by parasitic protozoa of the genus *Plasmodium*, which can lead to serious health complications. New antimalarial drugs are required to combat the rapid emergence and spread of multidrug-resistant parasites.

PdNPs synthesized from an aqueous extract of *Eclipta prostrata* leaves significantly inhibit the growth of *Plasmodium berghei* parasites in Swiss albino mice. They showed 78.13% good antimalarial activity against *P. berghei* parasites [137]. *Tinospora cordifolia*-PdNPs showed promising larvicidal activity against *Culex quinquefasciatus* and *Anopheles subpictus* [138]. The larvicidal effect of PdNPs was determined for larvae of *Aedes aegypti* and *Anopheles stephensi* mosquitoes [139]. This effect is likely attributed to the nanoparticles’ small size, which enables them to penetrate through the larval cell membrane or bind to DNA and membrane proteins, leading to denaturation of proteins or nucleic acids. Thus, the breakdown of cellular processes and eventual cell death can result from severe membrane depletion and proton motive force rupture [139].

An approach using *Butea monosperma*-PdNPs to determine the glucose content in dilute human serum samples was proposed. It has been shown that this colorimetric method is highly selective for determining glucose content [78]. Similarly, the method based on *Ginkgo biloba*-PdNPs has shown good accuracy in determining blood glucose concentration [98]. This colorimetric method also has high selectivity, a wide linear range, a low detection limit, and good accuracy in determining glucose concentration. In addition, these nanoparticles have peroxidase-like properties and typical Michaelis-Menten kinetics [98].

A method based on PdNPs was developed, allowing for the detection of hemoglobin over a wide range of concentrations, including low levels [140]. Palladium nanoparticles retained a clear reaction to hemoglobin in the presence of interfering substances (HSA, RNA, cytochrome C, transferrin, BSA, lysozyme, glucose, K^+^, Ca^2+^, Na^+^, Mg^2+^, CO_3_^2−^, and Cl^−^), indicating good selectivity of palladium nanoparticles in detecting hemoglobin.

PdNPs can penetrate the tumor through enhanced permeability and retention effects due to their relatively small size or through receptor-mediated binding using conjugated tumor-targeting ligands. Thus, PA (photoacoustic imaging) based on nanoparticles is suitable for detecting various types of tumors. In addition, lasers with different wavelengths can be used to obtain PA of nanoparticles that accumulate in the tumor due to the broadband absorption of nanoparticles. The nanoparticles were successfully demonstrated to be highly efficient for both in vitro photothermal therapy and in vitro photoacoustic imaging of tumors [100].

Chitosan oligosaccharide-coated biocompatible palladium nanoparticles effectively destroy tumors under the influence of 808 nm laser illumination at 2 W cm^−2^ power density. Moreover, they provide a good amplitude of photoacoustic signals, which facilitates the visualization of tumor tissues using a non-invasive photoacoustic tomography system. Such palladium nanoparticles are an ideal nanotheranostic tool for improved imaging and tumor therapy using a non-invasive near-infrared laser [141].

## 4. Toxicity

The biosafety profile of nanoparticles serves as a critical benchmark for evaluating their suitability for clinical applications. Therefore, it is crucial to understand the metabolism, pharmacological activity, pharmacokinetics, and biocompatibility of NPs, and their acute and long-term toxicity. Numerous in vitro studies have validated the biocompatibility of green PdNPs, highlighting their lack of toxicity to healthy cells in contrast to cancerous ones.

It is known that the toxicity of nanoparticles is related to their size, shape, surface charge, degree of agglomeration, and functional modifications. For example, nanoparticles smaller than 10 nm have the ability to penetrate cell membranes and tissue barriers, including the hematoencephalic and placental barriers. At the same time, the shape of the nanoparticles determines their interaction with cellular structures. For example, nanotubes and nanoplatelets have increased tissue penetration, which can cause localized damage, while spherical particles exhibit more predictable biocompatibility and uniform distribution within the body and are more frequently subject to phagocytosis. Biological synthesis is of particular interest because the coating molecules of extracts can reduce toxicity and enhance the biocompatibility of nanoparticles. Mechanisms of cellular toxicity include oxidative stress, effects on membrane transport and the immune system, and genetic effects. Furthermore, the use of nanoparticles can lead to a cumulative effect with undesirable consequences for the human body.

Hemocompatibility. To assess the biocompatibility of green PdNPs, a hemolytic analysis will be performed to calculate damage to human red blood cells (RBC). *Couroupita guianensis*-PdNPs have been shown to exhibit significantly lower hemolytic activity, indicating their biocompatibility and potential for use in clinical studies. The active bio-compounds present on the surface of these nanoparticles can significantly contribute to their enhanced biocompatibility [106]. Biogenic PdNPs from *Eryngium caeruleum* are human-friendly as they exhibit no cytotoxic effect against healthy RBCs, and the presence of various phenolic compounds from the plant extract as capping agents plays an important role in preventing the erythrocyte membrane oxidation, ultimately creating resistance to hemolytic activity [59]. *Sapium sebiferum*-PdNPs have no toxicity against RBCs at the tested concentrations (15, 30, 50, 70, 100, and 130 μg), as their toxicity is similar to that of the negative control [142]. Peripheral blood mononuclear cells (PBMCs), which make up 90% of immune cells and red blood cells, were used as a model system for assessing the toxicity of nanoparticles because nanomaterials first come into contact with blood cells shortly after administration. The MTT analysis showed that no cytotoxic effects or significant changes in the erythrocyte membrane were observed even at the highest dose of *Orthosiphon stamineus*-PdNPs compared with the control sample [102]. The capped PdNPs synthesized by *M. oleifera* are not involved in cell permeability for erythrocyte lysis [143]. Although the studies presented did not reveal any significant toxic effects of PdNPs on red blood cells, more extensive research is required in this area, as it is known that the smallest nanoparticles penetrate into the bloodstream, lymphatic system, and target organs (liver, spleen, lungs, and brain) [144]. Furthermore, for other metallic nanoparticles, higher toxicity of smaller nanoparticles than larger ones has been shown with respect to monocytes, macrophages, and lung epithelial cells [101]. The surface charge of nanoparticles may also be important. Positively charged nanoparticles interact more actively with cell membranes, which may enhance toxicity, whereas neutral or slightly negative surfaces demonstrate a milder interaction.

MTT analysis revealed that *M. oleifera*-PdNPs were selectively cytotoxic, inhibiting transformed A549 cells while showing no toxic effects on healthy peripheral lymphocytes (PLS) [70]. Similar data were demonstrated when studying palladium nanoparticles produced by *Urtica* plant extract [96]. PdNPs exhibited cytotoxicity against the MDA-MB-231 breast cancer, HT-29 colon cancer, and MiaPaCa-2 pancreatic cancer cell lines but showed no significant toxic effects on healthy L929 murine fibroblasts [104]. Tamarind seed gum-derived PdNPs were observed to significantly reduce the viability of MCF-7 cells. Their low cytotoxicity towards normal cells is attributed to the coating of a natural polysaccharide from tamarind seeds, which is rich in galactose, xylose, and glucuronic acid [145]. Hemicellulose-produced PdNPs decreased the HEK293, MDA-MB-231, HeLa, and Hep-G2 cell viability, and at the same time did not exhibit cytotoxic effects on normal cells, indicating that the synthesized PdNPs have excellent biocompatibility [146]. In J774 (murine macrophage cell line), cell viability was shown to be >95% at lower doses (25 and 200 mg/mL) and <50% at higher doses of *Parthenium hysterophorus*-PdNPs. Damage to cell membranes was also observed when taking higher doses, which could be due to modest levels of ROS generation, and the induction of apoptosis observed was moderate [79]. To date, considerable data have accumulated on the selective effect of nanoparticles on normal and cancer cells. However, this selectivity also requires more thorough verification, as it may be dose-dependent. Unfortunately, the literature currently lacks a fully proven mechanism for removing such nanoparticles from affected organs.

Studies on zebrafish embryos reveal that green-synthesized PdNPs, owing to their physical properties (shape, size, and surface area), exhibit no observable toxic effects. Teratogenic effects were found to be insignificant, and no deviations were observed at high concentrations [139]. The relative safety was demonstrated in marine shrimp. The mortality rate of *Artemia salina* was less than 50% when treated with various concentrations of biosynthesized PdNPs up to 300 micrograms, indicating their safety for biological use.

Concurrently, histological examination of zebrafish revealed distinct hepatic abnormalities following PdNP treatment, including hepatocyte rupture, vascular damage, erythrocyte accumulation, and sinusoidal congestion. These pathologies suggest an interruption of protein synthesis and the intracellular penetration of the nanoparticles. Exposure to *Annona squamosa*-synthesized PdNPs resulted in less severe liver damage compared to chemical PdNPs, with the extent of injury correlating with exposure duration. Observed histopathological features included binucleated cells, vacuolization, and red blood cell accumulation [145].

Since PdNPs are most often biosynthesized in a spherical form, their potential for medical application may be broader than that of other metallic nanoparticles. Capping with various biological molecules with antibacterial, anticancer, and other therapeutic properties can be an important regulator of their biocompatibility. Capping agents in this case can direct nanoparticles to specific cells or tissues, decreasing nonspecific interactions, providing a biocompatible shell, and mitigating toxicity and inflammatory reactions. However, it is important to understand that even biocompatible nanomaterials, if in excess concentrations or improperly modified, can cause toxic effects, including oxidative stress, inflammation, DNA damage, and apoptosis. However, understanding the mechanisms of cellular uptake, intracellular localization, and degradation is crucial to avoid associated negative exposures. Therefore, careful development and evaluation of the smart PdNPs’ targeted effects with targeted delivery, high biocompatibility, and subsequent degradation without harm to the human body are necessary.

## 5. Conclusions

In summary, green palladium nanoparticles constitute a unique, intriguing, and promising subject for future investigation and prospective medical applications. The diversity of biological entities that can serve as factories for producing these remarkable nanoparticles opens up a vast field for exploration in search of the most advanced, biocompatible, non-toxic nanoparticles for human cells produced under conditions that do not harm the environment. Biological molecules playing a crucial role in the formation and coating of palladium nanoparticles may be among the most important factors that determine their potential medical properties. Each method that utilizes biological materials as the basis for this synthesis, such as bacteria, fungi, algae, or plants, has its own advantages and should be selected based solely on the desired final properties of PdNPs. In our opinion, plants, particularly those with a well-documented history of medicinal use, represent a highly promising avenue for research. Furthermore, the potential for synthesizing nanoparticles from specific bioactive compounds warrants significant attention. Various phenolic compounds, alkaloids, terpenoids, and proteins, among others, can serve as an excellent platform for developing drugs. These compounds, according to their functional groups, can create electrostatic or spatial partitions around the surface of biologically mediated nanoparticles, not only to ensure their stabilization, but also to improve their catalytic properties, for example, under antibacterial action [108,147]. The application of new non-standard bio-platforms will expand the existing synthesis facilities base, and better understand the synthesis nature and interaction with human cells. For example, physiologically processed biomolecules contained in cow excrement have been proposed as a bio-factory for the synthesis of nanoparticles. Such PdNPs had high antibacterial activity against *Ps. aeruginosa* and *S. typhy*, and also had anti-oxidant properties [148].

The capacity of palladium nanoparticles to influence pathogenic bacteria, exhibit antioxidant properties, and demonstrate anticancer effects positions them as promising candidates for effective medical treatments. They can act as powerful therapeutic agents that can be further enhanced by conjugation with drugs and/or fluorophores and ligands for simultaneous diagnosis and targeted drug delivery to a cancer or infection site. For example, in an experimental rat infection model, *Allium sativum*-PdNPS alone and in combination with antibiotics showed improved elimination of MDR bacteria and improved epidermal regeneration [148]. These findings suggest that PdNPs offer promising antibacterial and antibiofilm agents and enhancers of antibiotic effectiveness and can be employed in wound dressings to prevent microbial infections [115].

The various strategies for obtaining and modifying palladium nanoparticles can provide many opportunities for the rational design of multifunctional nanoplatforms for cancer theranostics [149]. Combinations of palladium nanoparticles with chemotherapeutic drugs or prodrugs can be used as synergistic platforms for tumor treatment. There have already been some successes in the use of chemical PdNPs as contrast agents for imaging (photoacoustic imaging, single-photon emission computed tomography (SPECT), computed tomography (CT), and magnetic resonance imaging (MRI)). Based on the data obtained regarding palladium chemical nanoparticles, it can be inferred that biosensor platforms based on palladium nanoparticles may contribute to the identification of tumor subtypes and the formulation of personalized treatment plans [150]. Consequently, there is a reasonable assumption that bio-PdNPs could potentially play a similar role.

PdNP-based drug delivery nanosystems may also have great potential for successfully conjugating anticancer drugs and delivering them to a target cancer cell. Such nanocomplexes can significantly diminish cytotoxicity in non-cancerous cells, increasing the probability of successful treatment without causing side effects in healthy cells [151]. Positive results were detected in the human breast cancer cell treatment with a combination of the anticancer drug tubostatin and PdNPs synthesized using R-phycoerythrin [152]. This synergistic effect manifested itself in a significant inhibitory effect on cell viability compared to using only tubostatin or PdNPs and increased apoptosis due to the regulation of various cellular and biochemical changes [152]. This combination therapy aims to enhance the medical potential by using nanoparticles and existing anti-cancer medications. Additionally, the use of biomolecules with anti-tumor properties can both improve the effectiveness of nanoparticles and decrease their toxicity to healthy human cells. A distinct line of inquiry should be devoted to investigating the toxicity of green PdNPs with respect to normal human cells, both in vitro and in vivo. Despite the fact that numerous studies have demonstrated the absence of any toxic effects, it is crucial to thoroughly examine the mechanism of action and the potential risks to the human body associated with the use of such substances. It is important to evaluate both the activity level of antioxidant enzymes (SOD (Superoxide Dismutase), CAT, and LPO (Lipid Peroxidation)) as key biomarkers for oxidative stress, representing the balance between harmful ROS and the body’s antioxidant defenses, which reflect the adverse effects depending on the concentration and exposure duration, which is considered an important tool in the study of toxicity [153], and at the histological level—to perform tissue analysis of various organs (liver, spleen, lung, kidneys, and heart) to determine whether nanomaterials can cause tissue damage, inflammation, or pathological changes. The following can be highlighted as promising areas for reducing toxicity: surface modification and functionalization of nanoparticles by biological molecules with specified properties, control of size and morphology, development of biodegradable and biocompatible nanoparticles, and their use with targeted delivery to reduce systemic exposure.

Thus, obtaining non-toxic, biologically compatible PdNPs with desired therapeutic properties can expand the horizons of ideas about metal nanoparticles and their potential use in the treatment of various diseases.

## Figures and Tables

**Table 1:** PdNPs synthesized by bacteria.

Bacteria	Type of Synthesis	Size, nm	Shape	Ref.
*Calothrix*	intracellular	~10	spherical	[17]
*Desulfovibrio desulfuricans*, *Bacillus benzeovorans*	intracellular	0.2–8	icosahedrons	[18]
*Shewanella oneidensis*	on the outer membrane/perismatically	1–27	spherical	[21]
*Geobacter sulfurreducens*	extracellular	14–25	-	[22]
*Cupriavidus necator*, *Pseudomonas* *putida*, and *Paracoccus denitrificans*	intracellular	3–30	-	[24]
*Plectonema boryanum*	extracellular	1–30	spherical	[26]
*Desulfovibrio desulfuricans* *Escherichia coli*	perismatically/intracellular intracellular	20–30/1–5 1–3	-	[28]
*Cupriavidus metallidurans*	intracellular, on the cell surface, extracellular	≥26	cubical, rhombic dodecahedral, hexagonal, spherical, and rod- shaped	[31]

## Data Availability

No new data were created or analyzed in this study. Data sharing is not applicable to this article.
